# Dilution-to-Stimulation/Extinction Method: a Combination Enrichment Strategy To Develop a Minimal and Versatile Lignocellulolytic Bacterial Consortium

**DOI:** 10.1128/AEM.02427-20

**Published:** 2021-01-04

**Authors:** Laura Díaz-García, Sixing Huang, Cathrin Spröer, Rocío Sierra-Ramírez, Boyke Bunk, Jörg Overmann, Diego Javier Jiménez

**Affiliations:** a Microbiomes and Bioenergy Research Group, Department of Biological Sciences, Universidad de los Andes, Bogotá, Colombia; b Leibniz Institute DSMZ-German Collection of Microorganisms and Cell Cultures, Braunschweig, Germany; c Products and Processes Design Group, Department of Chemical and Food Engineering, Universidad de los Andes, Bogotá, Colombia; d Braunschweig University of Technology, Braunschweig, Germany; University of Bayreuth

**Keywords:** dilution to extinction, lignocellulose, microbial consortium, metataxonomic analysis, forest soil

## Abstract

The engineering of complex communities can be a successful path to understand the ecology of microbial systems and improve biotechnological processes. Here, we developed a strategy to assemble a minimal and effective lignocellulolytic microbial consortium (MELMC) using a sequential combination of dilution-to-stimulation and dilution-to-extinction approaches. The consortium was retrieved from Andean forest soil and selected through incubation in liquid medium with a mixture of three types of agricultural plant residues. After the dilution-to-stimulation phase, approximately 50 bacterial sequence types, mostly belonging to the *Sphingobacteriaceae*, *Enterobacteriaceae*, *Pseudomonadaceae*, and *Paenibacillaceae*, were significantly enriched. The dilution-to-extinction method demonstrated that only eight of the bacterial sequence types were necessary to maintain microbial growth and plant biomass consumption. After subsequent stabilization, only two bacterial species (*Pseudomonas* sp. and *Paenibacillus* sp.) became highly abundant (>99%) within the MELMC, indicating that these are the key players in degradation. Differences in the composition of bacterial communities between biological replicates indicated that selection, sampling, and/or priority effects could shape the consortium structure. The MELMC can degrade up to ∼13% of corn stover, consuming mostly its (hemi)cellulosic fraction. Tests with chromogenic substrates showed that the MELMC secretes an array of endoenzymes able to degrade xylan, arabinoxylan, carboxymethyl cellulose, and wheat straw. Additionally, the metagenomic profile inferred from the phylogenetic composition along with an analysis of carbohydrate-active enzymes of 20 bacterial genomes support the potential of the MELMC to deconstruct plant polysaccharides. This capacity was mainly attributed to the presence of *Paenibacillus* sp.

**IMPORTANCE** The significance of our study mainly lies in the development of a combined top-down enrichment strategy (i.e., dilution to stimulation coupled to dilution to extinction) to build a minimal and versatile lignocellulolytic microbial consortium. We demonstrated that mainly two selectively enriched bacterial species (*Pseudomonas* sp. and *Paenibacillus* sp.) are required to drive the effective degradation of plant polymers. Our findings can guide the design of a synthetic bacterial consortium that could improve saccharification (i.e., the release of sugars from agricultural plant residues) processes in biorefineries. In addition, they can help to expand our ecological understanding of plant biomass degradation in enriched bacterial systems.

## Abstract (precis)

The significance of our study mainly lies in the development of a combined top-down enrichment strategy (i.e., dilution to stimulation coupled to dilution to extinction) to build a minimal and versatile lignocellulolytic microbial consortium. We demonstrated that mainly two selectively enriched bacterial species (*Pseudomonas* sp. and *Paenibacillus* sp.) are required to drive the effective degradation of plant polymers. Our findings can guide the design of a synthetic bacterial consortium that could improve saccharification (i.e., the release of sugars from agricultural plant residues) processes in biorefineries. In addition, they can help to expand our ecological understanding of plant biomass degradation in enriched bacterial systems.

## INTRODUCTION

Currently, climate change and the exhaustion of petroleum have exacerbated the need to produce renewable-commodity chemicals (e.g., biofuels) from agricultural plant residues (1). However, one of the main obstacles in this bioconversion is the low efficiency of the saccharification step. This drawback is mainly attributed to the high complexity of plant cell walls (i.e., lignocellulose) (2, 3). The efficient release of monosaccharides from plant biomass requires the synergistic interaction of several enzymes (e.g., laccases, cellulases, xylanases, esterases, and monooxygenases) (4, 5). It has been suggested that enzyme cocktails produced by lignocellulose-degrading microbial consortia (LMC), instead of a single microbe, can be a promising strategy to optimize the saccharification process in biorefineries (6, 7). Previous reports have demonstrated the ability of LMC to secrete an array of different glycosyl hydrolases (GHs) after induction with agricultural plant residues (8, 9). In these studies, the authors suggested that synergism and metabolic versatility are key factors for efficient lignocellulose degradation. There are two main approaches to building LMC: (i) selection and diversity reduction from nature (i.e., top-down) (10) and (ii) design of synthetic communities with bacterial and/or fungal isolates (i.e., bottom-up) (11).

Soil ecosystems harbor a large diversity of microbes (e.g., bacteria and fungi) that can interact during the decomposition of plant biomass (12, 13). Thus, forest soils, which contain high quantities of decaying leaf litter and wood, are promising sources for lignocellulolytic microbes. The source of the inoculum and the type of substrate are the main drivers that determine the assembly, selection, and functionality of LMC (14–16). Moreover, it is known that the diversity, stability, and performance of any microbial consortium is determined by cooperative and/or negative interactions between their members (17, 18). The diversity within LMC positively correlates with high degradation rates but only until reaching an optimum point, denoted the minimal and effective consortium. Actually, a further increase in the species richness of the consortium could negatively affect the process due to enhanced competition (6, 19).

Previously, the effects of microbial diversity and synergism on lignocellulose degradation have been explored. For instance, Evans et al. (20) constructed bacterial synthetic communities with different levels of species richness and showed that lignocellulose degradation is directly related to diversity and the metabolic traits of particular species to degrade beta-glucan. Other studies have shown a positive effect between *Enterobacteriaceae* and *Sphingobacteriaceae* species growing on wheat straw (21, 22). Moreover, synthetic multispecies LMC have been developed. In this regard, a minimal and effective sugarcane bagasse-degrading consortium has been designed and characterized (19, 23). Similarly, Bohra et al. (24) built an LMC with five bacterial strains that can interact synergistically, resulting in enhanced cellulases production.

Recently, top-down enrichment strategies have been used to select low-diversity, efficient, stable, and resilient microbial communities with a desired metabolic function (25). To highlight, Gilmore et al. (10) designed a minimal microbial consortium able to produce methane from lignocellulose. We therefore assessed whether an innovative top-down enrichment strategy can guide the design of synthetic and effective LMC. Thus, we developed a combined selective approach (i.e., dilution to stimulation coupled to dilution to extinction) to build a forest soil-derived minimal and effective lignocellulolytic microbial consortium (MELMC). The goals were to (i) identify the minimum number of microbial species (i.e., bacterial sequence types) needed for the effective degradation of lignocellulosic biomass, (ii) develop a versatile MELMC capable of degrading different types of agricultural plant residues, and (iii) determine the range of plant polymers that can be depolymerized by the enzymes secreted by the MELMC.

## RESULTS

### Initial enrichment of LMC by the dilution-to-stimulation approach.

**FIG 1:**
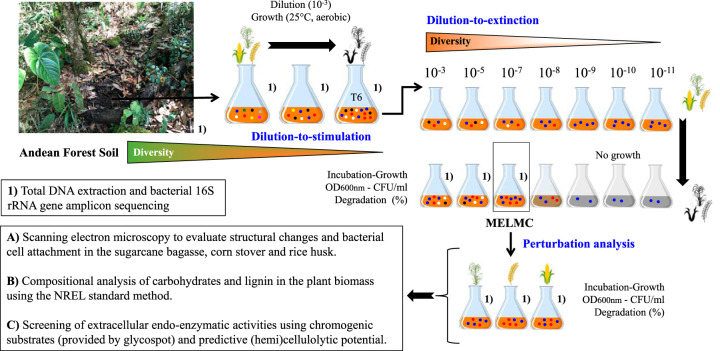
Schematic representation of the combined top-down enrichment strategy (i.e., dilution to stimulation coupled to dilution to extinction) used to develop the MELMC (minimal and effective lignocellulolytic microbial consortium). The inoculum source was Andean forest soils (photograph at the top left). For MELMC selection, a mixture of three agricultural residues (sugarcane bagasse [SCB], rice husk [RH], and corn stover [CS]) was used. Characterization of the MELMC was done after a perturbation analysis of these agricultural residues. See Materials and Methods for details.

**FIG 2:**
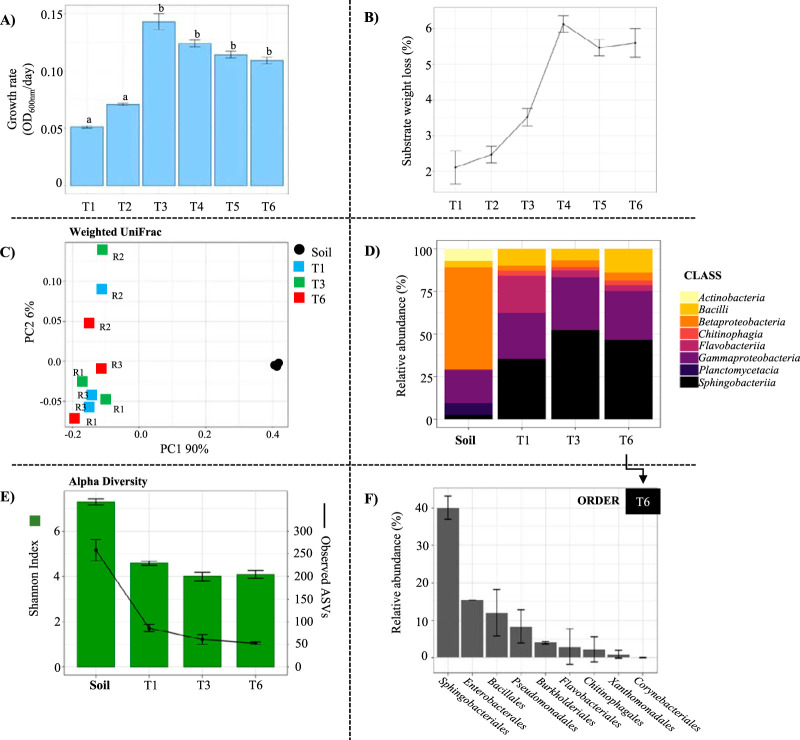
Dilution-to-stimulation approach used for the selection of an initial lignocellulolytic microbial consortium. (A) Microbial growth rate (optical density at 600 nm per day) along the sequential transfers (T1 to T6). Statistical differences are indicated with lowercase letters (*P ≤ *0.01 by ANOVA). (B) Average substrate weight loss (percent) after each transfer. Error bars represent standard deviations from three biological replicates. (C) Abundant bacterial classes (≥1% relative abundance) in the soil inoculum and T1, T3, and T6. (D) PCoA plot based on weighted UniFrac distances that shows the bacterial community similarity between the soil inoculum, T1, T3, and T6. (E) Observed amplicon sequencing variants (ASVs) and Shannon index values within the soil inoculum, T1, T3, and T6; all samples were rarified to 1,000 sequences. (F) Bacterial composition (at the order level) of the stabilized lignocellulolytic consortium at T6. Error bars represent standard deviations from three biological replicates.

As a first stage to develop an MELMC, the dilution-to-stimulation approach was carried out to enrich an initial LMC from Andean forest soil. Based on the assumption that an LMC selected on a specific agricultural plant residue is ineffective to degrade another one, the soil-derived community was cultivated in a mixture of three different agricultural plant residues (sugarcane bagasse [SCB], rice husk [RH], and corn stover [CS]) (Fig. 1). During the enrichment, the incubation time (to reach a bacterial population size of around 10^8^ CFU/ml) was reduced from 7 days in transfer 1 (T1) and T2 to 4 days after the last transfers (i.e., T3 to T6). These differences between transfers were significant (*P* value of <0.001), indicating a higher growth rate and consortium stabilization after T4 (Fig. 2A). Moreover, the weight loss of the substrate increased during the transfers, from a minimum of 2.0% ± 0.46% in T1 up to 6.0% ± 0.23% in T4. After T4, the substrate weight loss stabilized (Fig. 2B). Plate counting on potato dextrose agar (PDA) indicated that fungal populations were of low abundance (<10^3^ CFU/ml) at T6.

The bacterial 16S rRNA gene amplicon sequencing analysis generated ∼2.1 Gbp of high-quality reads and 908 amplicon sequence variants (ASVs). The taxonomic affiliation of ASVs revealed an increasing number of species belonging to the *Sphingobacteriia*, *Gammaproteobacteria*, and *Bacilli* classes along the sequential transfers (Fig. 2C). The families *Sphingobacteriaceae*, *Enterobacteriaceae*, *Pseudomonadaceae*, and *Paenibacillaceae* were significantly and differentially (log_2_-fold change [FC] of ≥2) abundant at T6 compared to the soil inoculum. Principal-coordinate analysis (PCoA) based on weighted UniFrac distances among microbial communities indicated that the soil inoculum was highly dissimilar from the consortium. Additionally, slight differences in the bacterial structural composition in biological replicate 2 (R2) compared to R1 and R3 were observed (Fig. 2D). Moreover, Shannon diversity values decreased from ∼7.2 in the soil inoculum to ∼4.0 in T3 and T6. Approximately 50 ASVs composed the LMC at T6 (Fig. 2E). The stabilized consortium contained high proportions of species belonging to the *Sphingobacteriales*, followed by the *Enterobacterales*, *Bacillales*, and *Pseudomonadales* orders (Fig. 2F). Regarding fungal communities, PCR amplification of the internal transcribed spacer 1 (ITS1) marker was negative (or below the detection limit) in all samples used for bacterial 16S rRNA gene amplicon sequencing, probably due to the low proportion of fungal DNA within the metagenomic DNA and/or biases in the extraction protocol.

### Selection and identification of an MELMC by the dilution-to-extinction approach.

**FIG 3:**
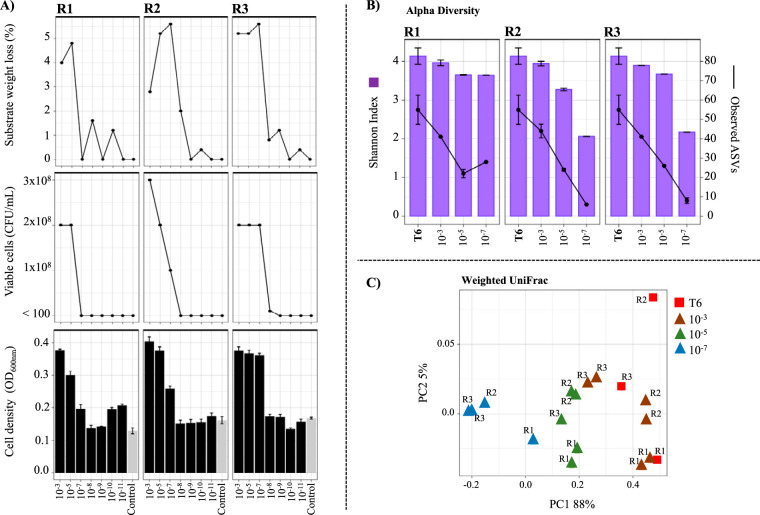
Selection of the minimal and effective lignocellulolytic microbial consortium (MELMC) using the dilution-to-extinction approach. (A) Microbial cell density (optical density at 600 nm), viable and cultivable bacterial cells (CFU per milliliter), and average substrate weight loss (percent) in three biological replicates (R1, R2, and R3) after incubation of the inoculated dilutions (10^−3^ to 10^−11^) created from microbial consortium T6, decreasing its diversity levels. The negative control did not contain a microbial inoculum. (B) Observed amplicon sequencing variants (ASVs) and Shannon index values within consortium T6 and after growth at 10^−3^, 10^−5^, and 10^−7^ dilutions in each biological replicate. (C) PCoA plot based on weighted UniFrac distances that shows the bacterial community similarity between T6 and the selected consortia after the growth at 10^−3^, 10^−5^, and 10^−7^ dilutions.

The selection of an MELMC was performed by dilution to extinction of the initial LMC (obtained at T6). In order to reduce the microbial diversity of this consortium, serial dilutions from 10^−3^ to 10^−11^ were done (Fig. 1 and 2). After incubation, significant differences (*P* value of <0.001) in cell densities (optical density [OD] at 600 nm) between 10^−3^, 10^−5^, and 10^−7^ dilutions and negative-control flasks were observed (Fig. 3A). Regarding viable cells, no microbial growth was observed after 10^−5^ (in R1) and 10^−7^ (in R2 and R3) dilutions (Fig. 3A). In R2 and R3, the MELMC was found in the 10^−7^ dilution with a bacterial growth value of between 1 × 10^8^ and 2 × 10^8^ CFU/ml. In addition, high values of substrate weight loss were obtained in the 10^−5^ (in R1) and 10^−7^ (in R2 and R3) dilutions (Fig. 3A). Based on the latter results, bacterial 16S rRNA gene amplicon sequencing was done with the 10^−3^, 10^−5^, and 10^−7^ dilutions in all biological replicates (R1, R2, and R3). A bacterial diversity-decreasing gradient was evident, where key lignocellulolytic species were lost at dilutions higher than 10^−7^. For the 10^−7^ dilution, the Shannon diversity values were ∼2.06 and ∼2.17 in R2 and R3, respectively (Fig. 3B). In these replicates, the ASVs decreased from ∼50 in T6 to ∼10 for the 10^−7^ dilution. R1 showed dissimilar values of alpha diversity. Moreover, weighted UniFrac distances revealed a change in the community structure from the initial LMC (at T6) to the MELMC. In addition, each biological replicate showed slight differences (e.g., R1 versus R2 and R3) (Fig. 3C).

### Taxonomic composition and stabilization of the MELMC.

**FIG 4:**
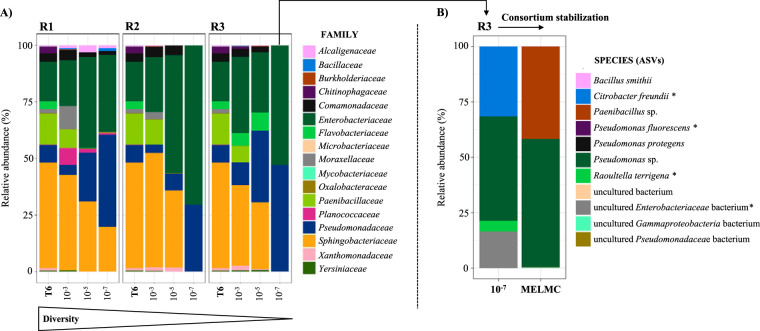
Bacterial composition (relative abundance) of the selected consortia after the growth of diluted samples from the T6 consortium (i.e., dilution-to-extinction approach). (A) Bacterial composition at the family level. (B) Bacterial composition at the species level (or ASVs) of the selected MELMC before (i.e., dilution of 10^−7^, replicate 3 [R3]) and after (i.e., two sequential transfers) the stabilization procedure. *, highlighted bacteria (Raoultella terrigena, Pseudomonas fluorescens, and Citrobacter freundii) that were completely depleted in the stabilized MELMC.

Based on the taxonomic affiliation of ASVs, differences in microbial composition across biological replicates and serial dilutions were observed. For instance, the *Flavobacteriaceae*, *Burkholderiaceae*, and *Moraxellaceae* families disappeared in the 10^−7^ dilution in all replicates (Fig. 4A). In R1, at the 10^−7^ dilution, the relative abundances of the *Alcaligenaceae*, *Comamonadaceae*, and *Sphingobacteriaceae* families remained low, while in R2 and R3, these taxa disappeared. In contrast, the *Pseudomonadaceae* family was abundant along dilutions (from ∼6% in T6 to ∼47% for the 10^−7^ dilution). Similarly, the *Enterobacteriaceae* family increased its relative abundance from T6 to 10^−7^ (e.g., ∼70 and ∼53% in R2 and R3, respectively) but was maintained stably in R1 between 10^−5^ and 10^−7^ dilutions (Fig. 4A).

The MELMC was selected in R3 at a dilution of 10^−7^ given that this community displayed higher values of growth and substrate weight loss (Fig. 3A). In this consortium, 11 ASVs were obtained, in which Raoultella terrigena (∼48%), *Pseudomonas* sp. (∼47%), Citrobacter freundii (∼31%), and an uncultured *Enterobacteriaceae* bacterium (∼16%) were the most abundant. During MELMC stabilization, some ASVs decreased their abundance and were depleted (highlighted with asterisks in Fig. 4B). In contrast, some species (or ASVs) found at very low abundances were highly enriched. For instance, the abundance of *Paenibacillus* sp. (99.78% nucleotide identity to *Paenibacillus* sp. strain T.M2R24) increased from ∼0.01% up to ∼42%, while *Pseudomonas* sp. (97.11% nucleotide identity to Pseudomonas protegens strain AL12) reached an abundance of ∼58%. The stabilized MELMC contains eight ASVs, capable of degrading ∼5.2% of a mixture of lignocellulosic biomass, where only two bacterial populations are highly abundant (>99%) (Fig. 4B).

### Degradation of agricultural plant residues and lignocellulolytic capacity of the MELMC.

**FIG 5:**
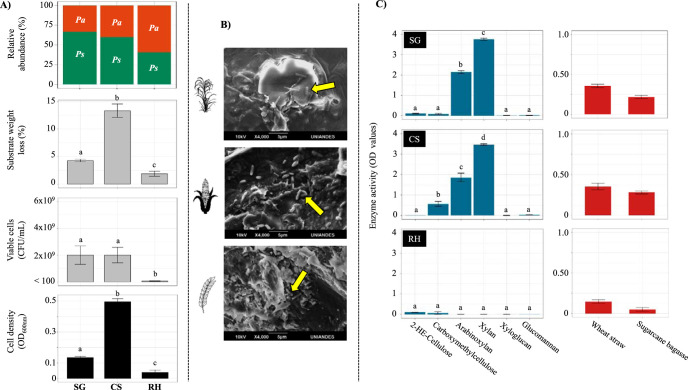
Characterization of the stabilized MELMC after its growth on individual agricultural residues (i.e., perturbation analysis). (A) Microbial cell density (optical density at 600 nm), viable and cultivable bacterial cells (CFU per milliliter), average substrate weight loss (percent), and relative abundances of *Pseudomonas* sp. (*Ps*) and *Paenibacillus* sp. (*Pa*) after growth of the MELMC on sugarcane bagasse (SCB), corn stover (CS), and rice husk (RH). Error bars represent standard deviations from three biological replicates, and statistical differences are indicated with lowercase letters (*P ≤ *0.01 by ANOVA). (B) Microbial cells attached to the agricultural residues (yellow arrows), evidenced by scanning electron microscopy. (C) Secreted endoenzymatic activities (OD values) on six chromogenic polysaccharide hydrogels (595 nm) (blue) and two insoluble biomass substrates (517 nm) (red) after MELMC growth on SCB, CS, and RH. Statistical differences are indicated with lowercase letters (*P ≤ *0.01 by ANOVA).

The ability of the MELMC to grow and deconstruct individual agricultural plant residues was evaluated. For all lignocellulosic biomass, significant differences (*P* value of <0.001) in microbial growth were obtained between them. The stabilized MELMC reached the highest values of cell density on corn stover (CS), followed by sugarcane bagasse (SCB) and rice husk (RH) (Fig. 5A). The MELMC showed a bacterial population density of around 2 × 10^9^ CFU/ml in CS and SCB (without significant differences between them [*P* value of >0.05]). These results were consistent with substrate weight loss, in which the percentage in CS reached 13.3% ± 1.22%, whereas that in RH was 1.8% ± 0.46% (Fig. 5A). The bacterial diversity/composition analysis showed that the stabilized MELMC did not change its composition after growth in each lignocellulosic biomass. However, some differences in the relative proportions of the key taxa were observed (Fig. 5A).

After MELMC growth, structural changes in each lignocellulosic biomass were evaluated by scanning electron microscopy. The images showed the presence of pores and fissures, probably caused by bacterial activity, in CS and SCB. In contrast, RH did not show any evident microstructural change (see Fig. S1 in the supplemental material). Moreover, many bacterial cells (i.e., bacillus) were found attached to the surfaces of CS, SCB, and RH (Fig. 5B). Based on the National Renewable Energy Laboratory (NREL) protocol (26), the percentages of lignin, cellulose, and hemicellulose were obtained in each substrate before and after MELMC growth (Table S1). The highest degradation percentages were obtained in CS (33.32% ± 1.65% for hemicellulose, 28.72% ± 1.65% for cellulose, and 17.91% ± 4.86% for lignin). In contrast, low degradation values were observed in RH (13.19% ± 0.79% for hemicellulose, 2.23% ± 0.59% for cellulose, and 1.64% ± 0.46% for lignin).

The ability to degrade plant polysaccharides was evaluated in the MEMLC metasecretome after growth in SCB, CS, and RH (16). Here, an array of chromogenic polysaccharide hydrogels and complex biomass were used. This method was useful to identify which type of substrates can be deconstructed by the action of secreted endoenzymes (i.e., those that can act on the polysaccharide backbone) (16, 27). The metasecretome of the MELMC, after growth in SCB and CS, showed the highest and most significant (*P* value of <0.001) enzymatic activities (i.e., absorbance values) on xylan and arabinoxylan (Fig. 5C). Interestingly, the MELMC secreted enzymes that could degrade other agricultural plant residues such as wheat straw, indicating its high versatility. Apparently, the MELMC metasecretome, retrieved after growth on CS, contained enzymes able to degrade carboxymethyl cellulose. However, significantly (*P* value of <0.01) lower enzymatic activities in the former chromogenic substrate were observed in SCB and RH (Fig. 5C).

### Predicted (hemi)cellulolytic potential of the MELMC.

**FIG 6:**
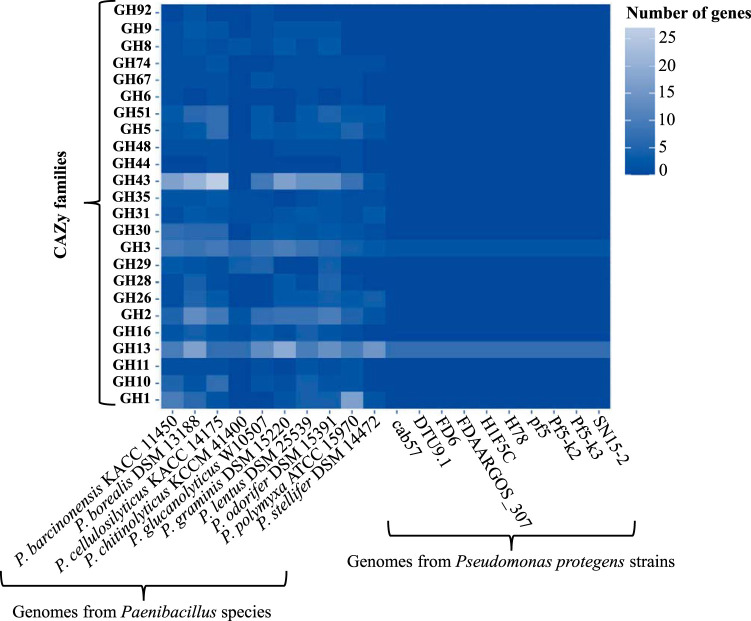
(Hemi)cellulolytic potential of 20 bacterial genomes (10 from *Paenibacillus* species and 10 from Pseudomonas protegens strains) based on the CAZyme annotation. The heat map was built using the number of genes associated with each glycosyl hydrolase (GH) family within each bacterial genome. The data were retrieved directly from the CAZy database (http://www.cazy.org/Genomes.html).

To unveil the (hemi)cellulolytic potential of the MELMC, two approaches were carried out. First, PICRUSt2 (Phylogenetic Investigation of Communities by Reconstruction of Unobserved States) software (28–30) was used to predict the metagenomic profile of the enriched consortia (Fig. S2). These results showed that the stabilized MELMC has the capacity to produce endoglucanases (EC 3.2.1.4), beta-glucosidases (EC 3.2.1.21), beta-galactosidases (EC 3.2.1.23), and alpha-mannosidases (EC 3.2.1.24). Second, the carbohydrate-active enzyme (i.e., CAZyme) profiles of 20 bacterial genomes (10 from *Paenibacillus* species and 10 from Pseudomonas protegens strains) were analyzed (Fig. 6). We suppose that the 20 selected bacterial types could be phylogenetically close to the key members of the MELMC, having similar metabolic potentials. In this analysis, the results suggested that *Paenibacillus* species contain a broad genomic potential to deconstruct plant polysaccharides compared with Pseudomonas protegens. In particular, high abundances of genes affiliated with CAZy families GH1, GH2, GH3, GH13, and GH43 were observed. In addition, genes that encode endoglucanases (e.g., GH5, GH9, GH16, and GH74) and endoxylanases (e.g., GH10, GH11, and GH30) were found only within the genomes of *Paenibacillus* species (Fig. 6).

## DISCUSSION

In previous studies, the enrichment of LMC has been successfully achieved using top-down approaches (14, 31–34). Here, a combined strategy was set up, coupling dilution-to-stimulation and dilution-to-extinction methods (25, 35), to assemble an MELMC capable of growing in a mixture of agricultural plant residues (Fig. 1). In the stimulation stage, the initial LMC became more effective in degrading plant biomass during sequential transfers (Fig. 2A and B). However, the substrate weight loss at T6 (∼5.5%) was lower than those for other reported LMC (15, 36). For instance, Zhang et al. (37) showed that a thermophilic LMC can degrade up to 20% of RH and corn stalk after 6 days of cultivation. The low degradation percentages observed in our consortium were attributed to the mixture of lignocellulosic biomasses and their highly recalcitrant nature, especially the RH. It is possible that the initial LMC (i.e., T6) grew using only CS or SCB. In addition, the absence of fungi and incubation conditions (i.e., short times, mesophilic temperature, and agitation) could negatively affect the weight loss values. Moreover, differences between the bacterial structures of the soil inoculum and the trained consortium (*P* value of <0.05 by permutational multivariate analysis of variance [PERMANOVA]) (Fig. 2D) suggested that a selective process, determined by the lignocellulose input in the culture flasks, drives community assembly. In this regard, Zegeye et al. (38) reported that substrate-driven soil community succession is mainly deterministic. Moreover, reductions of diversity and enrichment of *Sphingobacteriales* and *Enterobacteriales* species were evident at T6 (Fig. 2E and F). These fast-growing bacterial decomposers have been found at high abundances in other reported soil-derived LMC (14, 15, 21, 39), suggesting that they have the capacity to thrive in lignocellulose-enriched systems. The low proportion of fungal populations is attributed to the liquid and homogeneous cultivation conditions, in which short incubation times favor bacterial growth (32). Although fungi play key roles in lignocellulose deconstruction in nature, bacterial communities were highly abundant in our enrichment system, and they drive the deconstruction of plant biomass.

After the stimulation phase, a stable LMC composed of around 50 bacterial taxa was obtained (i.e., T6). However, the identification of hemicellulolytic species, lignin metabolizers, and sugar cheaters (i.e., species that consume sugars without contributing to the degradation process) can be very challenging at this stage (6). Hence, the dilution-to-extinction approach was carried out to select an MELMC, ensuring high lignocellulose degradation in a low-diversity microbial system. The reduction of diversity/complexity by the extinction method has been used to facilitate the isolation of heterotrophic soil microorganisms (40), to evaluate the impact of diversity in plant root colonization by fungi (41), and to analyze the transformation of pharmaceutical compounds in wastewater bioreactors (42). Interestingly, Kang et al. (25) used this dual approach (i.e., dilution to stimulation/extinction) to select a minimal bacterial consortium able to degrade keratin, allowing them to identify the key players in the degradation process. In our study, beta diversity analyses (Fig. 3C) allowed us to infer that stochastic processes and/or sampling effects, caused by extinction, could trigger different community assemblies in each biological replicate, and therefore, differences in microbial growth and degradation values were observed (Fig. 3A). Similarly, Kang et al. (25) reported these observations. Based on these results, we infer that a random reassembly of polymer-degrading bacterial populations could be more frequent in systems with reduced microbial loads and diversity. The occurrence of stochastic events could be relevant in systems with high dispersal under strongly selecting conditions (43), such as lignocellulose-enriched liquid cultures. Nevertheless, to confirm this statement, it would be necessary to carry out well-replicated experiments and null deviation analysis in order to obtain robust conclusions (44, 45).

Notably, a drastic change in the bacterial community composition was observed after a stabilization procedure (Fig. 4B). This step is imperative after the extinction method because the new microbial assemblages arrive at an unexploited environment and could gradually change to reach a stabilization state. Based on ecological theories about microbial adaptations to new environments (18, 46–48), some scenarios are proposed to explain the MELMC assembly. First, we hypothesize that *Enterobacteriaceae* and *Pseudomonadaceae* members are early-arriving species. They could reduce the availability of resources to late-arriving species (e.g., *Paenibacillus* sp.) following the niche preemption underlying priority effects. In this context, *Enterobacteriaceae* and *Pseudomonadaceae* members could be the fast colonizers (probably using the r reproductive strategy), given their high growth rates in unexploited, homogeneous, high-resource, and low-diversity environments (49). Interestingly, Goldford et al. (50) identified that soil-derived *Enterobacteriaceae* and *Pseudomonadaceae* species are well adapted to growth in liquid medium with a limiting and easy-to-consume resource (i.e., glucose). Second, we suggest that *Paenibacillus* sp. could use the K reproductive strategy, metabolizing complex substrates and dominating later stages of carbon decomposition during microbial succession (51). This member could have a high affinity for specific resources (e.g., xylose instead of glucose), turning it into a specialist player in a competitive and crowded environment. However, carbon uptake specialization, cross-feeding events, and lignocellulolytic synergistic processes in a partnership association with *Pseudomonas* sp. could reduce its competitive behavior within this microbial system. Third, we infer that *Paenibacillus* sp. has an advantageous position in lignocellulose-rich environments, compared to *Enterobacteriaceae* family members, due to its large enzymatic potential to deconstruct lignin and plant polysaccharides (23, 52).

The resilience of the selected MELMC was assessed by culturing (“perturbing”) the community on the individual agricultural plant residues used to develop it. Interestingly, the composition of the MELMC did not change after the perturbation analysis, confirming its stability and indicating that *Pseudomonas* sp. and *Paenibacillus* sp. are the key lignocellulolytic partners. Perturbation of other dilutions (e.g., 10^−5^) could generate new hypotheses and confirm the latter conclusion. The dominance of these bacterial species in the stabilized MELMC is similar to those that have been reported in other LMC. For instance, *Pseudomonas* and *Paenibacillus* species were highly abundant in a consortium that degrades RH and chlorophenols (53). Additionally, these two bacterial types are key members of a lignin-degrading consortium (54). Moreover, Woo et al. (55) isolated these bacteria from wet tropical forest soils, and they reported their high lignocellulolytic potential. In our study, high degradation values in CS and SCB were obtained (Fig. 5A). We suggest that the MELMC can grow better on these substrates due to the high availability of cellulose and hemicellulose compared to RH, in which a high percentage of lignin was observed (see Table S1 in the supplemental material). Based on our findings, the MELMC can deconstruct mostly the (hemi)cellulosic fractions of SCB and CS. In fact, cellulolytic, xylanolytic, and arabinoxylanolytic activities were detected in its metasecretome (Fig. 5C). The CAZyme profile of the 20 genomes from *Paenibacillus* species and Pseudomonas protegens strains suggested that *Paenibacillus* sp. could be the key (hemi)cellulolytic member within the MELMC, particularly due to it is potential to produce enzymes from the CAZy families GH5, GH9, GH10, GH11, GH16, and GH43 (23). Additionally, the analyzed genomes from *Paenibacillus* species contain genes that encode endoglucanases (e.g., GH5 and GH9) and endoxylanases (e.g., GH10 and GH11) (Fig. 6), suggesting that this consortium member could be responsible for the secretion of enzymes active on xylan, arabinoxylan, and carboxymethyl cellulose (Fig. 5C). Moreover, in the 20 bacterial genomes, 381 genes were affiliated with CAZy families GH1, GH2, and GH3 (Fig. 6), supporting the high abundance of beta-glucosidases and beta-galactosidases found in the PICRUSt2 prediction (Fig. S2). However, these results could be insufficient to reveal the actual enzymatic capability of the MELMC. Therefore, a metatranscriptomic and/or metaproteomic survey can give us a better picture of the expression/secretion of CAZymes in this microbial system (9, 23).

Soil-derived *Pseudomonas* species have been recognized as powerful ligninolytic microbes (34, 56, 57). In addition, they can secrete endoxylanases during wheat straw degradation processes (58, 59). Moreover, a strong correlation of cellulose degradation with the presence of *Paenibacillus* species has been reported in a synthetic LMC (19). Interestingly, a *Paenibacillus* sp. strain that is part of the former synthetic consortium can express endoglucanases, lytic polysaccharide monooxygenases, and endoxylanases along the degradation process for SCB (23). Based on our findings and these previous reports, we conclude that both lignocellulolytic partners within the MELMC have a large potential to transform lignin and deconstruct plant-derived polysaccharides. Specifically, we hypothesize that *Paenibacillus* sp. could be involved in cellulose and xylan degradation, with a high preference for metabolizing xylose. On the other hand, *Pseudomonas* sp. could play a pivotal role in lignin depolymerization and catabolism, consuming lignin-derived aromatic compounds and glucose as carbon sources.

Finally, the combined top-down enrichment strategy has been demonstrated to be an excellent approach to select a versatile MELMC, improving the understanding of ecological factors underlying plant biomass degradation. In addition, it can guide the design of a synthetic consortium that could be a prospective microbial system for agricultural waste valorization, especially in Colombia, where approximately 72 million tons of agricultural plant residues are produced per year and only ∼17% are reused (60). Further studies with the MELMC must be focused on (i) the isolation of the main bacterial members, (ii) confirmation of its lignocellulolytic capacity (e.g., enzymatic activities and genomic potential), and (iii) evaluation of the successive abundance dynamics in a two-strain microbial system using quantitative PCR (qPCR). In this regard, a coupled metaproteomic study could help us understand what are the key lignocellulolytic enzymes that are secreted and how they drive the interactions between the partners.

## MATERIALS AND METHODS

### Dilution-to-stimulation approach: development of the initial LMC.

Five randomly taken soil samples of 10 g were collected and mixed from a premontane wet Andean forest soil (0- to 10-cm depth) obtained in Gámbita (Santander), Colombia (5°56′18″N, 73°18′25″W). The microbial inoculum was prepared by adding 10 g of sampled soil and 10 g of sterile glass beads to 90 ml of mineral salt medium (MSM) [7 g/liter Na_2_HPO_4_, 2 g/liter K_2_HPO_4_, 1 g/liter (NH_4_)SO_4_, 0.1 g/liter Ca(NO_3_)_2_, 0.2 g/liter MgCl_2_ (pH 7.5)] in an Erlenmeyer flask. The flask was shaken for 20 min at 250 rpm, and 5 ml of the suspension was then withdrawn for DNA extraction. Aliquots (250 μl) of the soil suspension were added to Erlenmeyer flasks in triplicate, containing 25 ml of MSM with 1% lignocellulosic substrate consisting of sugarcane bagasse (SCB) (0.083 g), rice husk (RH) (0.083 g), and corn stover (CS) (0.083 g) (i.e., mixed in equal proportions). The liquid medium was supplemented with 25 μl of a trace element solution (2.5 g/liter EDTA, 1.5 g/liter FeSO_4_, 0.025 g/liter CoCl_2_, 0.025 g/liter ZnSO_4_, 0.015 g/liter MnCl_2_, 0.015 g/liter NaMoO_4_, 0.01 g/liter NiCl_2_, 0.02 g/liter H_3_BO_3_, 0.005 g/liter CuCl_2_) and 25 μl of a vitamin solution (0.1 g/liter nicotinic acid, 0.1 g/liter pyridoxal, 0.1 g/liter riboflavin, 0.1 g/liter thiamine, 0.01 g/liter biotin, 0.1 g/liter folic acid) (32) (Fig. 1). Agricultural residues were knife milled through a 1-mm screen and washed two times with distilled water and 70% (vol/vol) ethanol in order to remove soluble oligosaccharides. Materials were air dried in an oven at 50°C until constant weight and stored in 50-ml Falcon tubes at room temperature until use.

Culture Erlenmeyer flasks were incubated under aerobic conditions (130 rpm) at 25°C. After 7 days of growth, aliquots (250 μl) of the microbial suspension were transferred to 25 ml of fresh lignocellulose-containing medium to achieve a 10^−2^ dilution for the first two transfers. From transfer 3 (T3) to T6, aliquots (25 μl) of the microbial suspension were transferred to 25 ml of fresh lignocellulose-containing medium (dilution of 10^−3^) and incubated under the same conditions for 4 days. Two controls (i.e., without a substrate and without a microbial source) were also set up. At the end of each batch culture, flasks were gently shaken, solids were allowed to settle, and the liquid fraction was then removed by pipetting in order to determine the optical density (OD) at 600 nm. Cultures were filtered, and the dry weight of the substrate was quantified in order to calculate the percentage of weight loss, according to the formula proposed by de Lima Brossi et al. (15). Numbers of viable and cultivable cells (CFU per milliliter) were obtained by plate counting in R2A (Merck, Darmstadt, Germany) and potato dextrose agar (PDA) (catalog number CM139; Oxoid) at the end of each transfer. At each time of transfer, subsamples were collected and stored in 20% glycerol at −20°C. In order to evaluate significant differences in microbial growth along the transfers, analysis of variance (ANOVA) and a *post hoc* Tukey-Kramer test were performed using R software (R Core Team, 2008), with a confidence interval of 99% (α = 0.01). Data were tested for normality (Shapiro-Wilk) and subjected to the Levene test for homogeneity in the group variances.

### Dilution-to-extinction approach: selection and identification of the MELMC.

Triplicate 100-ml flasks with 1% lignocellulosic substrates and 25 ml of MSM (supplemented with trace element and vitamin solutions) were inoculated with 25 μl of 1:10 serially diluted preenriched consortia obtained by the dilution-to-stimulation approach. The dilution of the consortium was performed from T6, when the microbial community was stable, using a sterile 0.85% NaCl (saline) solution. Thus, we generated eight treatments: sterile medium (not inoculated) and 10^−3^, 10^−5^, 10^−7^, 10^−8^, 10^−9^, 10^−10^, and 10^−11^ dilutions in triplicate (Fig. 1). These flasks were incubated at 25°C for 5 days, with shaking at 130 rpm. At the end of the incubation, the cell density was determined (OD at 600 nm), along with microbial cell growth (plate counting on R2A and PDA) and the percentage of substrate weight loss. Stabilization of the MELMC was achieved after two transfers of the 10^−7^ dilution (replicate 3 [R3]) in MSM supplemented with 1% lignocellulosic substrates. Samples were taken from each dilution and stored in 20% glycerol at −20°C. In order to determine significant differences between the dilutions compared to the negative control (i.e., without microbial inoculum), ANOVA followed by *post hoc* Dunnett’s test was performed using R software (R Core Team, 2008).

### Perturbation analysis: evaluation of MELMC growth in individual plant agricultural residues.

In order to evaluate the versatility and the effect of single substrates on microbial growth, the MELMC was cultivated in MSM (supplemented with trace element and vitamin solutions) containing either 1% SCB, CS, or RH. Each flask was inoculated with 25 μl of the MELMC, and flasks were incubated at 25°C for 5 days with shaking at 130 rpm. This process was repeated two times in order to allow the stabilization of the microbial community. At the end of the final batch, the cell density (OD at 600 nm), microbial cell growth (plate counting in R2A and PDA), and percentage of substrate weight loss were measured (Fig. 1). In order to obtain statistical data, ANOVA and a *post hoc* Tukey-Kramer test were performed using R software (R Core Team, 2008), with a confidence interval of 99% (α = 0.01). Data were tested for normality (Shapiro-Wilk) and subjected to the Levene test for homogeneity in the group variances. Moreover, we determined the cell attachment and microstructural and/or morphological changes in the agricultural residues before and after MELMC growth using scanning electron microscopy. Samples were coated with gold using a Denton Vacuum Desk II sample metallizer. The images were acquired at a 10-kV accelerating voltage. In order to evaluate the proportions of cellulose, hemicellulose, and lignin on the agricultural residues before and after MELMC growth, we used the compositional analysis reported by the NREL (26). The absorbance reading for acid-soluble lignin was taken at 320 nm for CS and 240 nm for RH and SCB using a UV-visible (UV-Vis) spectrophotometer (model T80+; PG Instruments) with quartz cuvettes. The monosaccharides produced by acid hydrolysis were analyzed by high-performance liquid chromatography (HPLC) (Agilent 1100 series), using an Aminex HPX-87H column (Bio-Rad, Philadelphia, PA, USA) and a refractive index detector with Milli-Q water as the mobile phase at 0.6 ml/min, with a column temperature of 80°C. Samples of 20 μl were automatically injected. The calibration curves were determined using the following standards: glucose, xylose, arabinose, cellobiose, and mannose. Degradation percentages were calculated as the relation between the contents of each polymer before and after MELMC treatment. Correction of data was performed using the solid and dry weight values of each substrate in order to determine accurate weight loss percentages.

### Determination of polysaccharide-degrading enzymatic activities in the MELMC metasecretome.

Enzymatic activities of the MELMC were evaluated after growth (25°C for 5 days, with shaking at 130 rpm) in each agricultural residue. Semiquantification of the secreted endoenzyme activities was carried out using a new generation of versatile chromogenic substrates (27) according to the instructions of the manufacturer (GlycoSpot IVS, Farum, Denmark). Briefly, 25 ml of each culture was filtered through cloth, and the supernatant was recovered. Enzymatic activities were determined by employing 2-hydroxyethylcellulose (2-HE-cellulose), carboxymethyl cellulose, arabinoxylan, xylan, xyloglucan, glucomannan, wheat straw, and sugarcane bagasse as the substrates. The chromogenic substrates were available in a 96-well filter plate format. They were activated by the addition of 200 μl of activation solution and incubation for 10 min at room temperature. The activation solution was removed by centrifugation (2,700 × *g* for 10 min), and the plate was washed twice with 100 μl of water to remove the stabilizer. Subsequently, 200 μl of the culture supernatant was added to each well, and samples were incubated for 24 h at 25°C at 130 rpm. After the transfer of the supernatant into the collection plate, absorbance values were determined at 595 nm (blue) for purified polysaccharide hydrogels and at 517 nm (red) for insoluble complex biomaterial substrates, using a plate reader (Multiskan Go; Thermo Scientific). The negative control was MSM (supplemented with trace element and vitamin solutions) containing 1% SCB, RH, or CS without the microbial inoculum. Statistical comparisons between the absorbance values were performed using one-way ANOVA and a *post hoc* Tukey-Kramer test.

### DNA extraction and bacterial 16S rRNA amplicon sequencing.

DNA from the dilution-to-stimulation approach, the dilution-to-extinction approach, and the perturbation analysis was extracted in triplicate using the DNeasy UltraClean microbial kit (Qiagen, Hilden, Germany) according to the instructions of the manufacturer. Sequencing of the bacterial 16S rRNA gene was performed by using Illumina MiSeq technology (2- by 300-bp paired-end reads) at the Leibniz Institute DSMZ. The hypervariable V3-V4 region was amplified and sequenced using the primers 341F (5′-CCTACGGGNGGCWGCAG-3′) and 785R (5′-GACTACHVGGGTATCTAATCC-3′) (61), according to the instructions for Illumina according to the 16S metagenomic library preparation protocol (https://emea.support.illumina.com/downloads/16s_metagenomic_sequencing_library_preparation.html). Sequencing raw data were processed using Quantitative Insights into Microbial Ecology (QIIME2) software (62). Raw paired-end reads were joined using VSEARCH (63). The deblur pipeline (64) was used to perform quality control and correct reads to obtain amplicon sequence variants (ASVs). After processing, 1,035 ASVs were observed, ranging from 1,682 to 65,609 sequences across 40 samples. The data were rarified to 1,000 reads per sample for comparisons between samples and to calculate diversity metrics. Multiple alignments of ASVs were built using MAFFT (65). FastTree (66) was used to construct a phylogenetic tree based on the MAFFT alignment. ASV taxonomic assignment was done using the SILVA rRNA gene database (67). To obtain a better ASV taxonomic classification, the Basic Local Alignment Search Tool (BLAST) was employed against the NCBI RefSeq database. In addition, QIIME2 was used to generate relative abundances of ASVs, alpha and beta diversity metrics (including Shannon’s index), observed ASVs, and weighted UniFrac distances. PERMANOVA was used to perform multivariate analysis of beta diversity metrics. The differential relative abundance was evaluated using the DESeq2 plug-in in the Phyloseq package using R studio software.

### Prediction of the metabolic potential by PICRUSt2.

The ASV abundance table and representative 16S rRNA gene sequences generated by QIIME2 were used as the inputs to predict the metabolic potential (i.e., functional abundance values), along the MELMC selection, using PICRUSt2 (Phylogenetic Investigation of Communities by Reconstruction of Unobserved States) software (28). Based on previous studies (29, 30), the relative proportions of 23 enzyme-encoding genes (KEGG orthology identifiers) predicted to be involved in (hemi)cellulose degradation were evaluated. The predicted 16S rRNA gene copy numbers were used to normalize the relative abundances values. The nearest sequenced taxon index (NSTI) values were calculated to evaluate the accuracy and reliability of metagenome prediction. To improve the predictions, we used NSTI scores lower than 1. Phylogenetic placement of reads was done using the EPA-NG (68) and Gappa (69) tools. The Castor R package (70) was used to predict the gene family abundances. The KEGG orthology (KO) inference was done with the MinPath package (71).

### Predictive carbohydrate-active enzyme profile.

A descriptive analysis of the carbohydrate-active enzyme (CAZyme) profiles of 20 bacterial genomes was used as a proxy to predict the (hemi)cellulolytic potential of the MELMC. The genomes were selected based on the 16S rRNA gene taxonomic affiliation (i.e., best hits) of the two most abundant ASVs in the stabilized MELMC (*Paenibacillus* sp. and Pseudomonas protegens). The CAZyme profile of each genome was retrieved from the CAZy database website (http://www.cazy.org/Genomes.html). Based on previous studies, 24 glycosyl hydrolase (GH) families predicted to be involved in (hemi)cellulose degradation were selected (16, 23). Finally, a heat map was built with R studio software using the number of genes associated with each GH family that were found in each bacterial genome.

### Data availability.

The bacterial 16S rRNA gene amplicon sequencing data obtained in this study have been deposited under NCBI Sequence Read Archive (SRA) accession number SRR11908309.

## Supplementary Material

Supplemental file 1
