# Supplementary material for: Dilution-to-Stimulation/Extinction Method: a Combination Enrichment Strategy To Develop a Minimal and Versatile Lignocellulolytic Bacterial Consortium
Source: Appl Environ Microbiol. 2021 Jan 4;87(2):e02427-20. doi: 10.1128/AEM.02427-20 (PMC7783344; doi:10.1128/AEM.02427-20)
Supplement: Supplemental file 1 [file AEM.02427-20-s0001.pdf]

SUPPLEMENTARY FIGURES

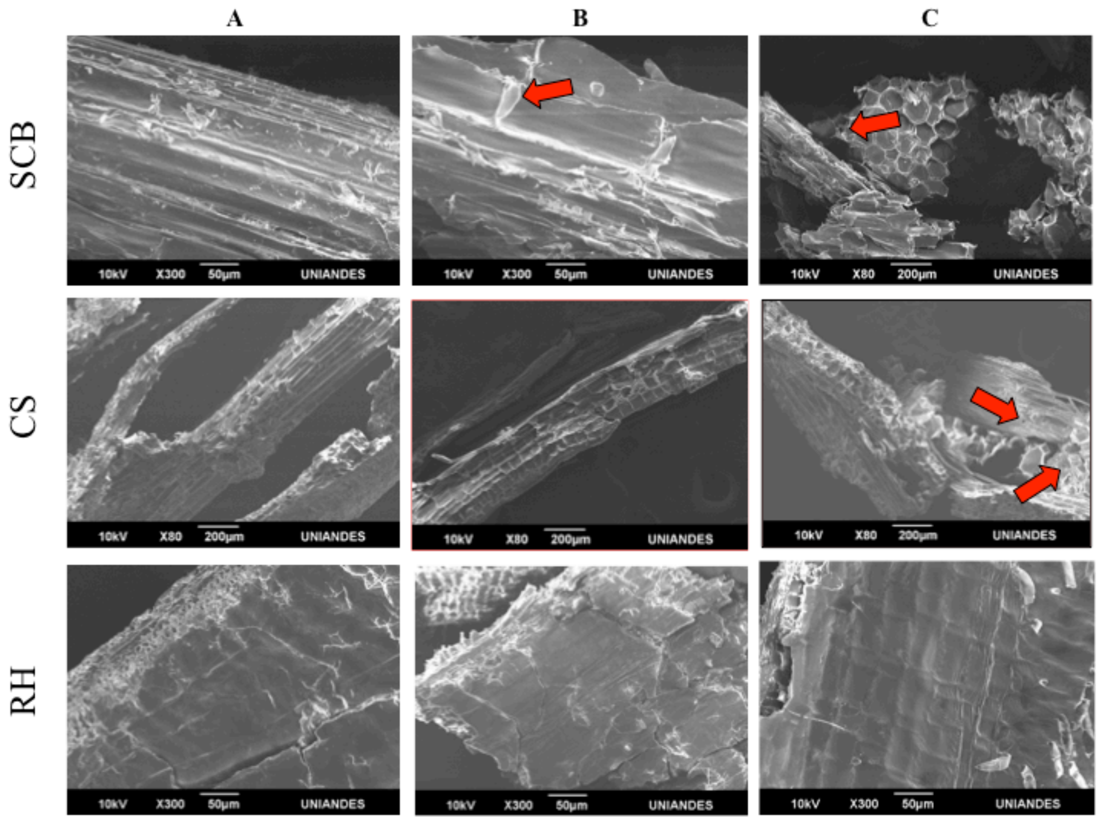

Fig S1. Microstructural changes in sugarcane bagasse (SCB), corn stover (CS) and rice husk (RH) after the perturbation analysis. A). Negative control. B and C) Scanning Electron Microscopy (SEM) micrograph of lignocellulosic biomass. Red arrows indicate the microstructural changes (e.g. pores) in the lignocellulosic biomass.

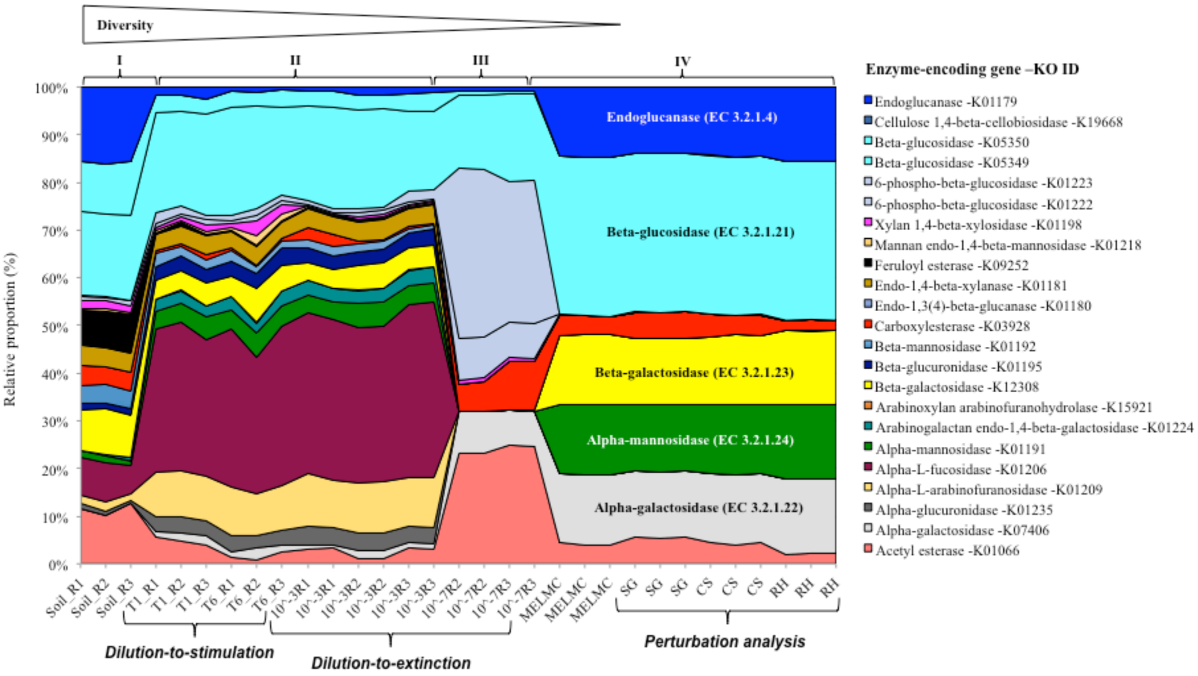

Fig S2. Predictive (hemi)cellulolytic profile along the MELMC selection using the PICRUSt2 software. X-axis shows samples: soil inoculum, T1, T6 (dilution-to-stimulation), 10^{-3}, 10^{-7} only R2-R3 (dilution-to-extinction) and the stabilized MELMC grown on the mixture of substrates, sugarcane bagasse (SG), corn stover (CS) and rice husk (RH). Y-axis shows the relative abundance (%) of 23 enzymes-encoding genes (KEGG Orthology IDs) involved in cellulose and hemicellulose degradation. The results showed four different profiles (denoted in roman numbers) along the MELMC selection procedure. The first one corresponds to the soil inoculum, the second encompasses samples T1, T6 and 10^{-3}, the third was obtained in the dilution 10^{-7} and the fourth was the stabilized MELMC grown in SCB, CS and RH.

SUPPLEMENTARY TABLES

Supplementary Table 1. Relative content (%) of cellulose, hemicellulose and lignin in sugarcane bagasse, corn stover and rice husk before (raw) and after the MELMC grown (treated). Each value is the mean of three biological replicates.

Agricultural residue	Cellulose (%)	Hemicellulose (%)	Lignin (%)
Raw sugarcane bagasse	30.10 ± 0.061	18.28 ± 0.000	22.80 ± 0.008
Treated sugarcane bagasse	27.18 ± 0.017	14.34 ± 0.083	22.19 ± 0.324
Raw corn stover	32.28 ± 0.501	20.40 ± 0.000	19.52 ± 0.006
Treated corn stover	27.50 ± 0.069	16.25 ± 0.240	19.16 ± 1.239
Raw rice husk	18.42 ± 0.000	14.99 ± 0.000	28.10 ± 0.003
Treated rice husk	18.13 ± 0.125	13.12 ± 0.131	27.83 ± 0.107
